# Plasmonic enhanced photocatalytic activity of Ag/TiO_2_ tube-in-tube fibers†

**DOI:** 10.1039/d2ra07207f

**Published:** 2022-12-15

**Authors:** Siyuan Zhang, Zewen Sun, Yue Zhou, Wenshu Chen, Qianhui Wu, Jianhua Sun, Leiming Lang

**Affiliations:** School of Chemistry and Chemical Engineering, Institute of Advanced Functional Materials for Energy, Jiangsu University of Technology Changzhou 213001 Jiangsu Province China sunjh@jsut.edu.cn; Excellent Science and Technology Innovation Group of Jiangsu Province, Nanjing Xiaozhuang University Nanjing 211171 China langleiming@njxzc.edu.cn

## Abstract

Ag nanoparticle was found to significantly enhance the photocatalytic activity of self-organized TiO_2_ nanotube structures. Herein, novel Ag/TiO_2_ tube-in-tube fibers have been prepared by a facile electrospinning technology and calcination process. Employed as the photocatalyst, the composite could efficiently catalyze the photodegradation of the model organic pollutant, rhodamine B under visible light irradiation, exhibiting a superior photocatalytic activity than the undoped TiO_2_ tube-in-tube fibers. This enhanced activity has been ascribed to plasmonic characteristics of Ag nanoparticles, which promote the light absorption and charge transfer feasibility. The simple, low-cost and green fabrication route of the composite provides a novel means for preparing similar materials, holding great promise for wider application in the future.

## Abstract 2

Ag nanoparticle was found to significantly enhance the photocatalytic activity of self-organized TiO_2_ nanotube structures.

## Graphical abstract

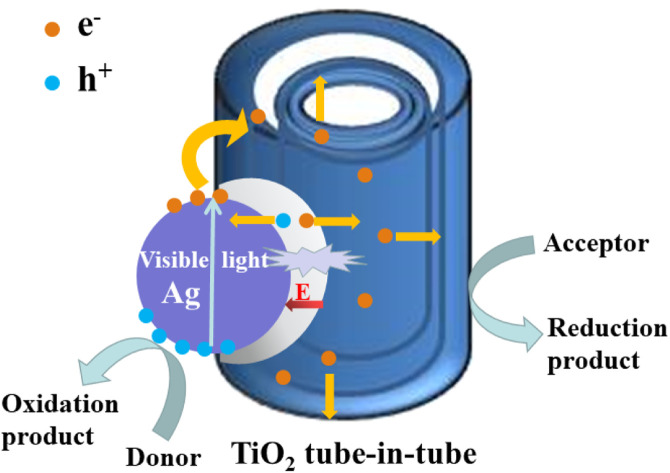

## Introduction

Photocatalysis, which utilizes the inexhaustible solar energy to drive chemical and energy processes, serves as a fascinating way to resolve the daily increasing environmental and energy crisis.^1–5^ Ever since the first report on photocatalytic water splitting pioneered by Fujishima and Honda,^6,7^ this technique has aroused wide interests and received considerable research globally.^8–12^ The operating mechanism for photocatalytic events is rather complex, but is generally accepted to initiate with the “charge-separation” step, during which the photo-responsive material absorbs photons and produces the electron–hole pair.^13–16^ These highly reactive species then triggers the specific following steps. For instance, both components could lead to the formation of hydroxyl radical (·OH) in aqueous condition, which is considered as a powerful and green oxidant for the degradation or removal of organic pollutants.^17–20^

The efficiency of a photocatalytic event is related to many factors including the light source, the photocatalyst, the reaction condition and so on. Among them, the property of photocatalyst is considered most important since it decides the efficiency of light harvesting, charge separation and the reaction of photo-generated carriers. In this regard, transition metal oxide (TMO) nanomaterials, which possess unique characteristics like special electronic structures, rich valence states and large surface area have attracted much attention.^21–25^ Numerous TMOs (ZnO, TiO_2_, *et al.*) have been used in photocatalytic applications like solar cells, pollutant degradation and photo-induced chemical bond formation.^26–33^

Born with a wide bandgap of 3.0–3.2 eV, TiO_2_ renders itself an ideal occasion for photo-induced charge separation and the subsequent photochemical events. In together with its low cost, chemical stability and being environmentally-friendly, these features forge TiO_2_ the most popular TMO material used in photocatalysis^24–29^ or thermal catalysis,^30,31^ and so on. Notably, numerous approaches have been developed to expand the light responsive wavelength range of TiO_2_, including heteroatom doping, sensitization with organic dyes, heterojunction with narrow-bandgap semiconductors and hybridization with noble metal nanostructures. The involvement of plasmonic metals (Au, Ag, Pt, Pd, Cu) within the matrix of TiO_2_ not only enables its response toward visible light due to their characteristic plasmonic resonance within this range, but also facilitates the charge separation and transfer processes vital to photocatalytic reactions.^34–43^ Thus, this method has been applied for the fabrication of many high-efficiency photocatalytic systems. For instance, Yang's group^44^ placed the Au nanoparticles into TiO_2_ arrays, resulting Au@TiO_2_ plasmonic films exhibited enhanced catalytic activity toward oxygen reduction reaction under simulated sunlight irradiation. Giri's group^39^ reported the role of surface plasmons and hot electrons for Ag@TiO_2_ nanorods on the photocatalytic decay under visible light. Some other studies also reflected the aspects of efficient LSPR hot electron injection for Ag/TiO_2_ catalysts.^38–41,45^

Besides, the surface property, microscopic morphology and grain size of TiO_2_ also play important roles in determining its photocatalytic activity. According to previous findings, the defects on TiO_2_ surface could trap the photo-induced carriers, inhibiting their recombination and increasing their utilization efficiency.^46,47^ On the other hand, the morphology of TiO_2_, including its high-index facet exposure, porosity and hierarchical structure could influence the separation and transfer kinetics of the charge carriers. These structural factors could be tuned by varying the synthetic condition of TiO_2_, which have also been widely studied.^48–50^ Various kinds of TiO_2_ nanostructures have been synthesized with controllable morphologies, tunable sizes and adjustable surface properties using different synthetic methods.^27–33,48–50*a*^

As we all know, Rhodamine B is a kind of organic dyes and widely applied to printing and dyeing industry. But it is a serious risk to the environment once mixed into natural water due to its high carcinogenicity and mutagenicity and low biodegradability. Therefore, the high effective photocatalysts are desired for the degradation of Rhodamine B in the industrial waste water. Here in this work, we report a simple route to synthesize silver nanoparticle incorporated TiO_2_ tube-in-tube fibers (ATTFs) through the simple electrospinning technique and calcination process. Compared with the undoped TiO_2_ tube-in-tube fibers (TTFs) and the catalyst of Ag loaded commercial TiO_2_ powder (ACTP), the as-prepared ATTFs exhibits much enhanced photocatalytic performance for dye photodegradation under visible light irradiation, holding promising potential for practical application like pollutant degradation.

## Results and discussion

**Fig. 1:**
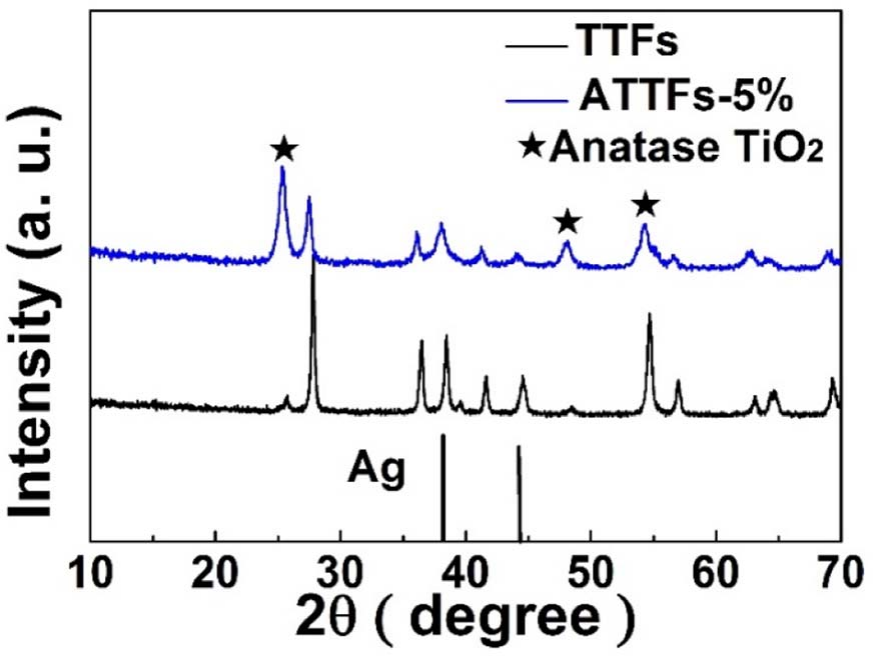
XRD patterns of TTFs and ATTFs-5%. The stars indicate the corresponding anatase TiO_2_ diffraction peaks.

X-ray diffraction (XRD) measurement was applied to analyze the chemical composition and crystalline structure of the samples. As shown in Fig. 1, the XRD patterns of ATTFs-5% and pure TiO_2_ without Ag exhibit the characteristic diffraction peaks of both rutile (27.4°, 36.5°, 56.6°) and anatase (25.4°, 48.0°, 54.6°) titanium dioxide, suggesting the mixture of two crystal forms. In addition to the signal of TiO_2_, the ATTFs-5% also depicts the diffraction peaks of Ag centered at 38.1° and 44.3°, which correspond to the (111) and (200) planes of the cubic phase Ag (JCPDS file 04-0783), respectively. The intensity increase of rutile titanium dioxide diffraction peaks for the ATTFs-5% maybe ascribe to the introduction of Ag to TiO_2_ tube-in-tube, which changed the TiO_2_ phase structure partly during calcination. The coexistence of both rutile and anatase crystal forms of TiO_2_ is considered beneficial toward the photocatalytic reactions similar to the commercial P25 photocatalyst.

**Fig. 2:**
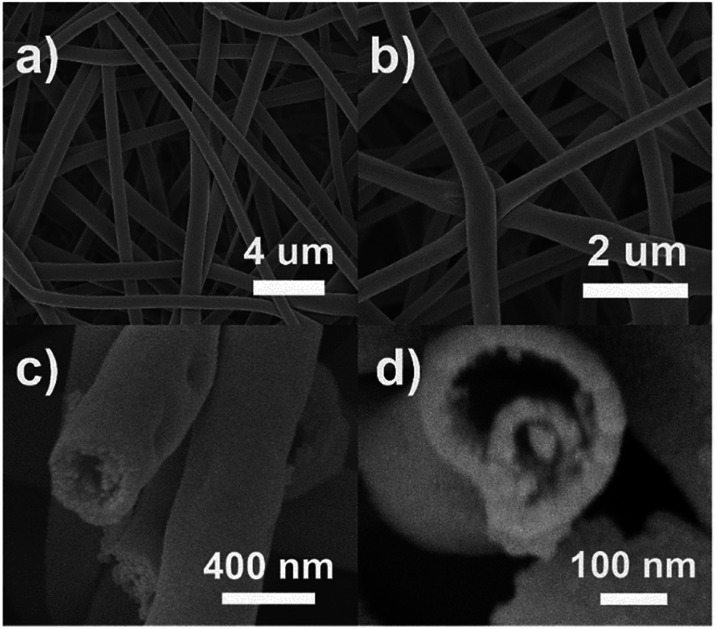
The typical SEM images of the ATTFs-5% (a) before calcination and (b) after calcination. (c) Locally magnified side-view and (d) high resolution top-view SEM images of one tube-in-tube after calcination.

**Fig. 3:**
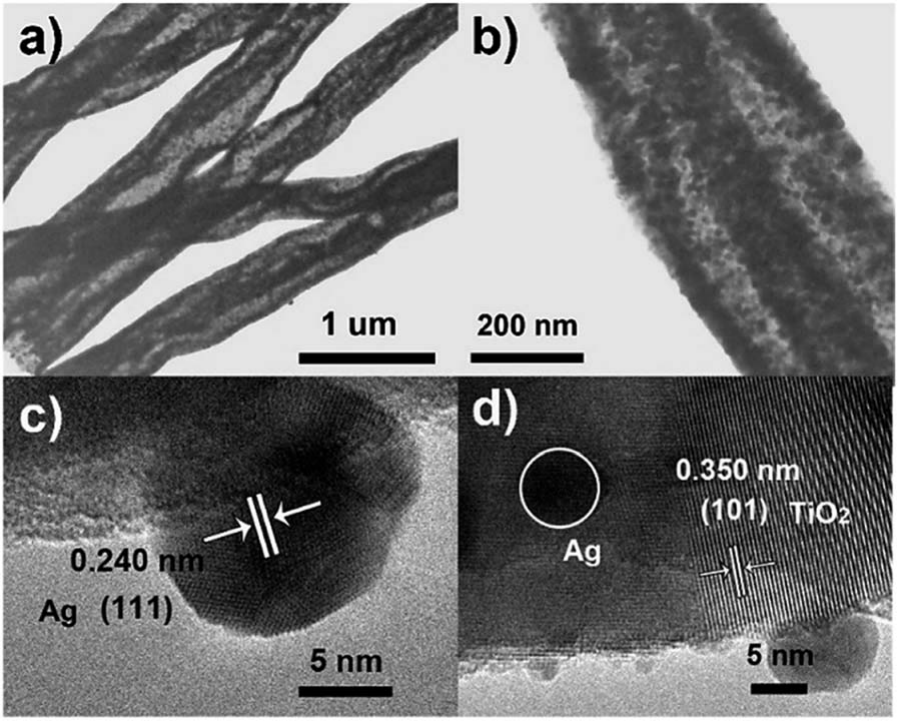
(a) Low-magnification and (b) magnified TEM and (c and d) HRTEM images of the ATTFs-5%.

The microscopic morphology of the composite is further disclosed by SEM and TEM. Fig. 2a–d depicts the SEM images of the ATTFs-5% at different stages and with different magnifications. The precursor fibers before calcination (Fig. 2a) are uniform with a diameter of around 1 μm and a rather smooth surface. The calcination treatment brings significant size and morphological change to the sample with a diameter of about 400 nm as shown in Fig. 2b. A local magnification toward one single fiber (Fig. 2c and d) clearly reveals its tube-in-tube structure with obvious hollow cavities inside the fiber. This hollow structure is also manifested in the TEM image (Fig. 3a and b). The magnified TEM result (Fig. 3b) also substantiates the TiO_2_ tube-in-tube is composed of small nanoparticles and has the obvious porous structure, which is believed to be the surface-anchored silver nanoparticles. The high-resolution TEM (HRTEM) image (Fig. 3c) centered at a single Ag nanoparticle on the edge of the fiber, which portrays a Ag nanoparticle diameter of ∼10 nm. The estimated interplanar spacing is 0.240 nm, corresponding well to (111) planes of cubic Ag. Due to thickness, the lattice fringe of the inner tube could not be analyzed. On the outer tube, only one set of lattice fringes of 0.350 nm could be discerned which corresponds to the (101) planes of anatase TiO_2_ (Fig. 3d). Further HRTEM image (Fig. S1†) showed the existence of rutile TiO_2_ with the (110) lattice plane. These results match well with those of XRD, which indicates the incorporation of cubic Ag within the mixed two crystal forms of TiO_2_.

**Fig. 4:**
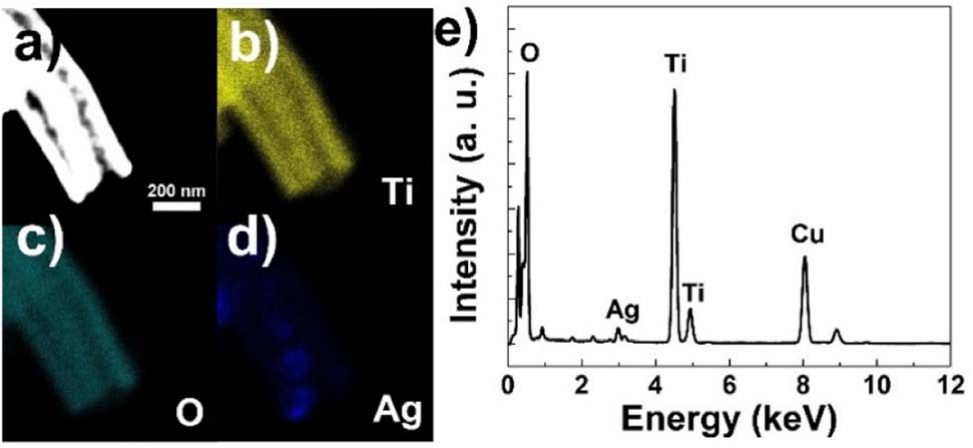
(a) Dark field TEM image, EDS mapping results of (b) Ti, (c) O, (d) Ag elements and (e) EDS pattern of ATTFs-5%.

The elemental composition and distribution of the fiber were probed using energy dispersive X-ray spectroscopy (EDS). Fig. 4b–d depicts the elemental mapping results of Ti, O and Ag corresponding to the dark field TEM image (Fig. 4a), respectively. It could be seen that Ti and O elements distribute homogeneously and uniformly on both the inner and outer tubes (Fig. 4b and c). On the other hand, Ag signal presents a smaller area and is mainly detected from inside the fiber, coinciding well with the TEM results. As shown in Fig. 4e, the EDS pattern of the area shown in Fig. 4a evidences the co-existence of Ti, Ag, O elements, while the Cu signal comes from the copper grid used as TEM substrate. The atom percent of Ag are is about 4.8%, matching well the feed ratio. Thus, based on the above results, the silver-nanoparticle incorporated tube-in-tube morphology of the ATTFs is explicitly confirmed.

**Fig. 5:**
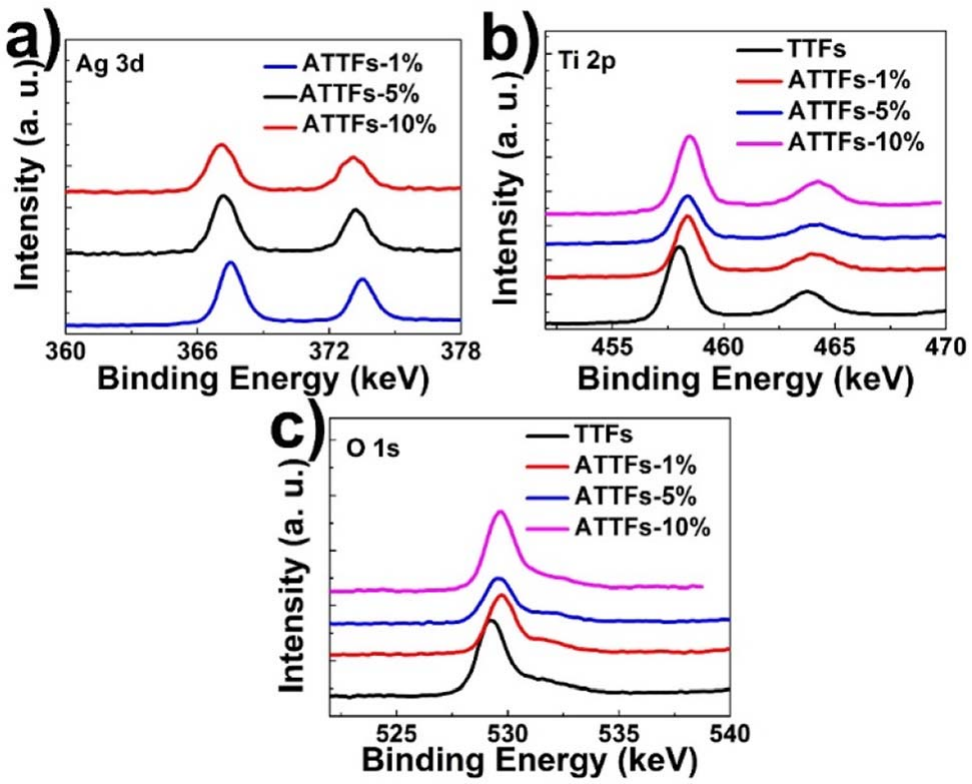
XPS survey spectrum of ATTFs with different silver contents: (a) Ag 3d, (b) Ti 2p and (c) O 1s.

XPS measurement was performed to probe the chemical status of the elements on the surface the composite. Fig. 5 depicts the XPS survey spectrum of ATTFs-5%, which clearly shows the characteristic O 1s, Ti 2p and Ag 3d signals. The XPS fine spectrum of Ag 3d (Fig. 5a, black curve) reveals the 3d_5/2_ and 3d_3/2_ peaks at 367.5 and 373.5 eV, respectively with a peak separation of 6 eV, characteristic of the metallic silver (Ag^0^). Compared with the signal of bulk Ag (368.3 eV for 3d_5/2_ and 374.3 eV for 3d_3/2_), both peaks shift toward lower binding energies (B. E.), which is ascribed to the electronic interaction between two components and a partial electron transfer from the TiO_2_ backbone into the Ag nanoparticle. An increase in the negative B. E. shift could be observed with increasing the Ag content in the composite, indicating stronger extent of interaction in high Ag loading samples. This feature is also reflected in the Ti 2p spectrum. As shown in Fig. 5b, the incorporation of silver nanoparticle leads to a significant positive shift of Ti 2p_3/2_ and 2p_1/2_ peaks compared with pure TiO_2_ tube-in-tube, which demonstrates a lower electron density of the Ti atoms in the ATTFs. The reverse shifts of the peaks for Ag 3d and Ti 2p illustrate a strong interaction between the metallic Ag and TiO_2_, which can effectively promote charge transfer and prohibit the recombination of excited electrons and holes at the Ag and TiO_2_ interface. This may ascribe that the Ag particles on the surface of TiO_2_ will act as electron acceptors and contribute to separate the photoexcited electron–hole pairs. The variation trend of O 1s XPS spectrum is similar to that of Ti 2p.

**Fig. 6:**
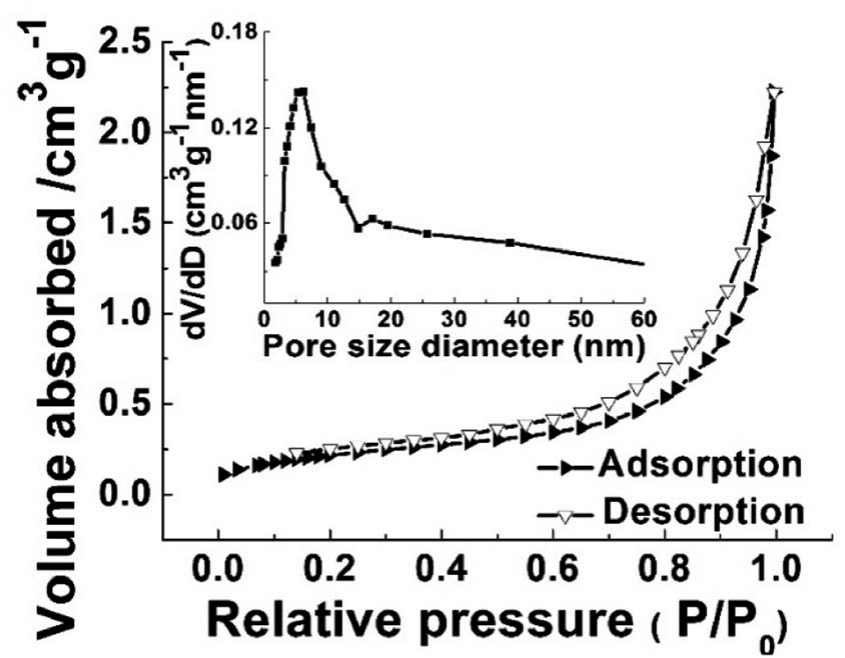
N_2_ adsorption/desorption isotherms and pore size distribution plot (inset) of ATTFs-5%.

To gain insights on the specific surface area and pore size distribution of ATTFs-5%, Brunauer–Emmett–Teller (BET) N_2_ adsorption–desorption measurement was conducted. As shown in Fig. 6, the ATTFs-5% exhibits the typical type IV isotherm plot with an apparent hysteresis loop, indicative of a mesoporous microstructure. The BET analysis outputs a specific surface area value of ∼55 m^2^ g^−1^. Meanwhile, the pore size distribution plot presents a sharp peak at ∼5.8 nm with a broad shoulder, reflecting that the less uniform distribution of pores in the tube wall, which might be ascribed to the disordered alignment of TiO_2_ and silver nanoparticles within the fiber. Nevertheless, the porous microstructure with a large surface area still contributes to the photocatalytic reactions, providing sufficient active sites for substrate adsorption and transformation.

**Fig. 7:**
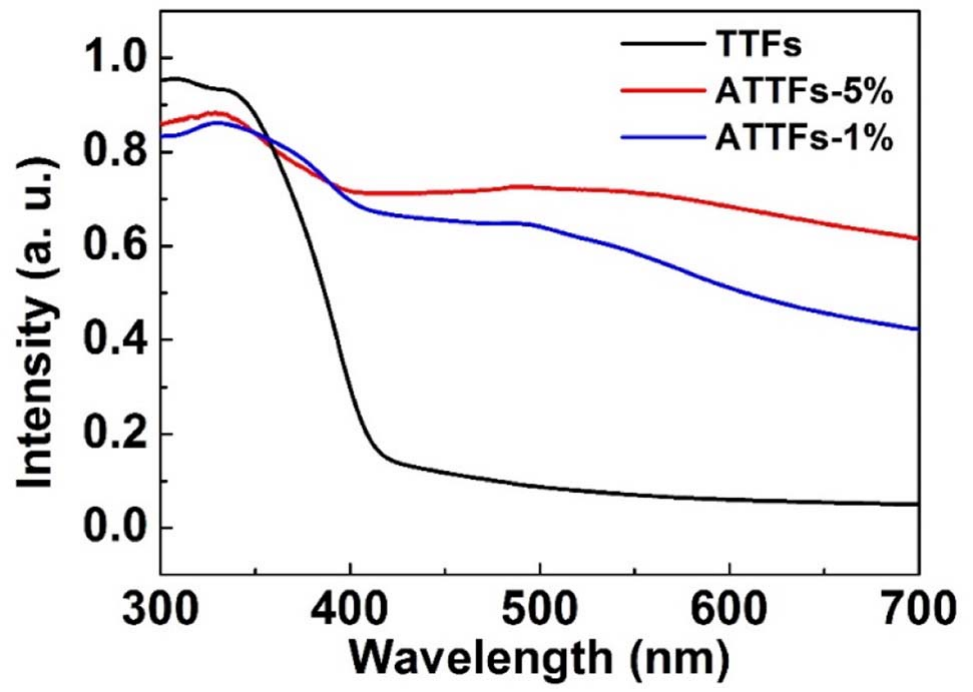
UV-vis diffuse reflectance spectra of TTFs and ATTFs.

Meanwhile, UV-vis diffuse reflectance spectroscopy was applied to investigate the UV-vis absorption behavior of the as-prepared samples. As shown in Fig. 7, the TTFs without Ag exhibits poor absorption in the visible region with an apparent absorption edge at ∼420 nm. The incorporation of Ag nanoparticles significantly increases the absorbance toward visible light with a broad band centered ∼500 nm, which arises from the strong LSPR resonant absorption of Ag nanostructures in this range. Reasonably, the ATTFs-5% exhibits stronger absorption than the ATTFs-1% counterpart due to the involvement of more Ag nanoparticles.

**Fig. 8:**
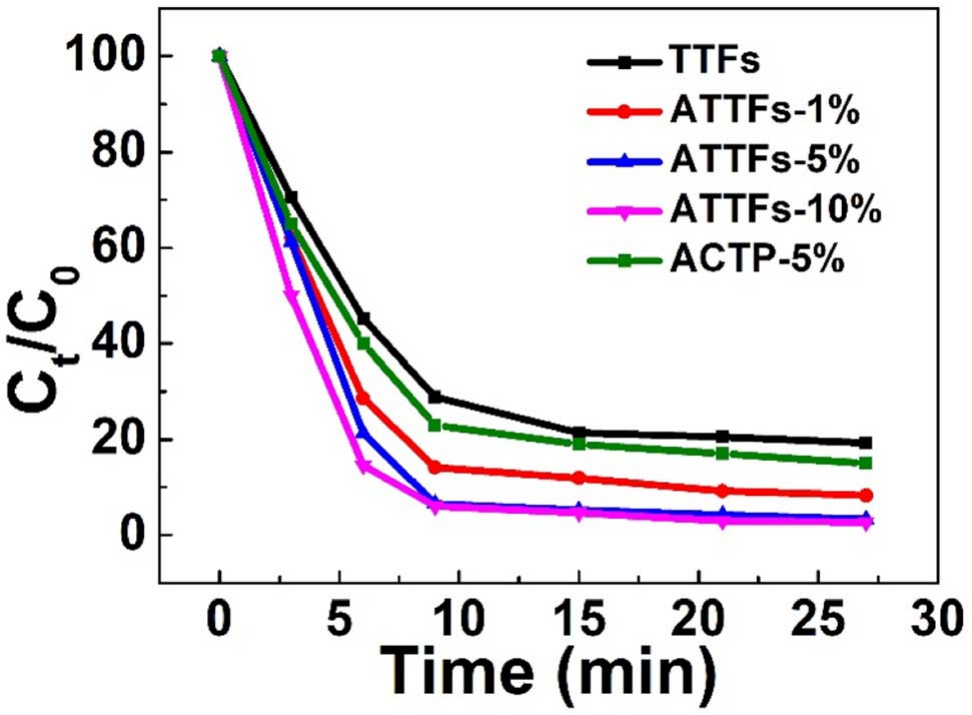
Time-dependent degradation efficiencies of different materials plotted using the normalized absorbance.

The photocatalytic performance of the as-prepared materials was investigated taking visible-light driven photocatalyzed degradation of RhB as a model. RhB is an organic dye with characteristic absorption, which can monitor the photocatalytic reaction. As shown in Fig. S2–S6,† both the TTFs and ATTFs exhibit considerable activity toward the photodegradation of RhB, manifested by the gradual decrease in 536 nm absorbance with the extension of illumination time. Notably, the systems catalyzed by ATTFs-5% and ATTFs-10% achieved an almost complete removal of RhB after 33 min, as evident from their negligible absorbance at 536 nm, while the two systems for TTFs and ATTFs-1% still present a substantial RhB absorption after the same reaction time. This fact signifies the surpassing catalytic activity of the ATTFs-5% and ATTFs-10%, which is more straightforwardly reflected in the time-dependent degradation efficiency curve as shown in Fig. 8. Compared from the curve slopes, it could be seen that the RhB degradation kinetics grows with increasing the silver content in the material, which could be ascribed to the promoted absorption toward visible light due to the involvement of silver nanostructures. To eliminate the UV light effect, the photocatalytic performance of ATTFs-5% was investigated under visible light irradiation by using UV filter (Fig. S7†), which showed a similar result to that of irradiated under the xenon light source. The ATTFs with higher silver content absorbs more photons due to the LSPR effect of silver nanostructures, which promotes the charge-separation process and in turn facilitates the formation of hydroxyl radical responsible for the degradation of RhB. On the contrary, the visible-light utilization efficiency of pristine TiO_2_ or 1% ATTFs is poor, impeding the generation of charge-carriers and results in the lower catalytic efficiency. Based on the experimental finding that the ATTFs-5% exhibited similar catalytic efficiency to its 10% counterpart, 5% is considered an ideal value for silver content in the ATTFs, reaching a balance between high photocatalytic activity and relatively low cost. For comparison, the photocatalytic performance of ACTP was investigated, which showed the low catalytic efficiency due to the small surface area and lack of multitubular-like structure.

**Fig. 9:**
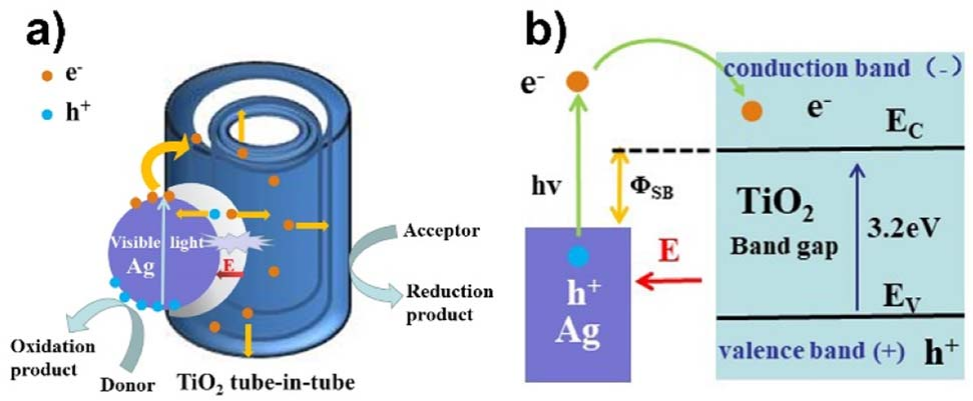
(a) Proposed photocatalytic mechanism and (b) schematic energy band diagram of the Ag/TiO_2_ heterostructure.

The enhanced visible light photocatalytic activity of the ATTFs attributes to the synergetic effect of strong coupling between Ag and TiO_2_ and band bending, which promotes efficient charge transfer at the interface. Fig. 9 shows a schematic of the TiO_2_ tube-in-tube coupled with an Ag NP. The presence of Ag NP on TiO_2_ causes a Schottky barrier (*φ*_SB_) with a space charge region in the TiO_2_ region, which gives rise to an internal electric field (*E*) from the TiO_2_ to Ag.^36^ The electric field forces the separation of the generated electron–hole pairs in the space charge region in the opposite directions and prevents their easy recombination in TiO_2_.

With the illumination of visible light, the Ag NP on the surface of TiO_2_ absorbs light, which drives the electrons of conduction band of Ag being excited and generates electrons with high kinetic energy at the Ag surface. The energy of these excited electrons is greater than the Schottky barrier height (*hν* > *φ*_SB_) and can escape easily from the Ag surface and are collected by the TiO_2_ tube-in-tube, since TiO_2_ is a good electron-accepting metal oxide with the high density of states in its conduction band. Furthermore, the separation of these electrons is accelerated between the TiO_2_ region and the Ag surface with the assist of the internal electric field (*E*). The produced electrons and holes can react with the O_2_ and H_2_O molecules adsorbed on the surface of ATTFs, which achieves highly active super oxide and hydroxyl radicals. Eventually, the dye can be degradated efficiently by these radicals under the visible light irradiation.

It should be noted that although the silver nanoparticles act as the photo-responsive component which generates the hot carriers, one cannot simply conclude that keep increasing silver content could bring even better photocatalytic performance. The catalytic efficiency of the Ag/TiO_2_ nanofiber is influenced by many factors, among which the density of Ag NPs on the surface of TiO_2_ may be taken in priority. When the silver content is too high, the numerous separated electrons and holes can recombine together again, which might lead to a decrease in catalytic efficiency. Moreover, when the surface of TiO_2_ is covered with too many silver NPs, the mesoporous structure of the material might be perturbed and its active surface area might decrease for the adsorption of RhB. Therefore, an optimal silver content of 5% exists for the Ag/TiO_2_ composite, neither too high nor too low shall renders the best photocatalytic activity, which is demonstrated in Fig. 8. Meantime, the multitubular-like structure of TiO_2_ is beneficial for the enhancement of photodegradation efficiency because of the relative large surface area and high visible light utilization efficiency, which endured the multiple reflection inside TiO_2_ tube-in-tube.

## Conclusions

In this work, a simple “sol–gel” method coupled with the electrospinning technique was applied for the fabrication of a highly efficient composite photocatalyst, the Ag/TiO_2_ nanofiber with a tube-in-tube microscopic morphology. The distinctive microstructure of the TiO_2_ backbone together with the densely distributed silver nanoparticles inside the fiber cavities have been characterized under various techniques. The involvement of plasmonic silver nanoparticles enables the visible response of TiO_2_ due to their resonant absorption within this range. As a result, the Ag/TiO_2_ composite exhibited greatly enhanced photocatalytic activity toward the degradation of RhB under visible irradiation, rendering itself a promising candidate for practical water pollutant treatment. Meanwhile, the green and facile fabrication route of Ag/TiO_2_ composite also provides useful insights toward the preparation of similar materials, its widespread use for the preparation of high-performance photocatalytic systems is strongly prospected.

## Experimental section

### Synthesis of Ag/TiO_2_ tube-in-tube fibers (ATTFs)

Ag/TiO_2_ tube-in-tube fibers (ATTFs) were prepared by a simple electrospinning technique, followed by a subsequent calcination process. Typically, 2.5 g of PVP-K30 was dissolved in 5 mL of ethanol at room temperature under stirring. After adding 2 mL of acetic acid to the above transparent solution, silver nitrate and tetrabutyl titanate was introduced the mixed solution was kept stirring till complete dissolvation, where the Ag content is 1%, 5% and 10% of the TiO_2_ mass for different samples. The prepared spinning solution was filled into a 10 mL tip glass syringe with an 18 gauge 90° blunt-end steel needle. Electrospinning was carried out at 16 kV using a high voltage supply, with the feeding rate of 0.3 mL min^−1^ by a syringe pump. The nanofibers were formed on the electrically grounded tin foil with the tip-foil distance set at ∼15 cm. After electrospinning, the nanofibrous mat was carefully peeled off from the foil, putted into a ceramic plate and placed in the tube furnace. The subsequent calcination was performed in air condition at 500 °C for 2 h at a heating rate of 2 °C min^−1^, which formed the final product termed as 1% Ag/TiO_2_ tube-in-tube nanofibers (ATTFs-1%). 5% Ag/TiO_2_ (ATTFs-5%) and 10% Ag/TiO_2_ nanofibers (ATTFs-10%) were prepared under similar procedures, except that the amount of silver nitrate in the spinning dope was different. For comparison, the catalyst of Ag loaded commercial TiO_2_ powder (ACTP) was obtained by the reduction of Ag^+^ in the mixed water solution contained commercial TiO_2_ powder, where the content of Ag was controlled to 5%.

### Photodegradation of dye

To evaluate the photocatalytic activity of the as-prepared nanofibers, photocatalytic dye degradation was performed with RhB chosen as the target compound. In a typical photodegradation experiment, 0.1 g of the sample was added in a quartz vessel which contains 400 mL of RhB aqueous solution (0.025 mmol L^−1^). The dispersion was magnetically stirred in dark for 30 min at room temperature, allowing the adsorption of dye on catalyst surface to reach equilibrium. The vessel was then loaded onto the photocatalytic set-up, which is equipped with a xenon light (350 W) source and a circulating water system to instantly remove the heat generated by the light source. The system was then exposed to continuous light irradiation, during which the photocatalyst catalyzed the degradation of RhB. To explore the catalytic efficiency, supernatants of the reaction system were taken at an interval of 3 min. These supernatants were centrifuged, followed by an absorption spectral analysis toward the filtrates, through which the remaining concentration of RhB at certain reaction stages could be derived from the characteristic absorption peak of RhB by an UV-visible spectrophotometer (LAMBDA-35, US). Comparable reactions were also carried out on Ag/TiO_2_ with different Ag contents.

## Author contributions

S. Zhang and Z. Sun contributed equally.

## Conflicts of interest

There are no conflicts to declare.

## Supplementary Material

RA-012-D2RA07207F-s001
