# Supplementary material for: Plasmonic enhanced photocatalytic activity of Ag/TiO2 tube-in-tube fibers
Source: RSC Adv. 2022 Dec 15;12(55):35820–6. doi: 10.1039/d2ra07207f (PMC9752503; doi:10.1039/d2ra07207f)
Supplement: RA-012-D2RA07207F-s001 [file RA-012-D2RA07207F-s001.pdf]

Electronic Supplementary Material (ESI) for RSC Advances.
This journal is © The Royal Society of Chemistry 2022

Supporting Information for

Plasmonic Enhanced Photocatalytic Activity of Ag/TiO_2 Tube-in-tube Fibers

Siyuan Zhang,[a,b] Zewen Sun,[b] Yue Zhou,[b] Wenshu Chen,[b] Qianhui Wu,[b] Jianhua Sun,[*a] Leiming Lang[*b]

[a]*School of Chemistry and chemical Engineering, Institute of Advanced Functional Materials for Energy, Jiangsu University of Technology, Changzhou 213001, Jiangsu Province, China.*

[b]*Laboratory of Advanced Functional Materials of Nanjing, Nanjing Xiaozhuang University, Nanjing, 211171, P. R. China.*、

Experimental Section

Reagents

Polyvinyl pyrrolidone (PVP-k30), silver nitrate, tetrabutyl titanate, rhodamine B (RhB), ethanol and acetic acid of analytical reagent purity were purchased from Sigma-Aldrich Chemical Co. Ltd. All reagents were used as received without further purification.

Apparatus

The chemical composition, crystalline structure and microscopic morphology of the samples were characterized by X-ray diffraction (XRD, Shimadzu XD-3A X-ray diffractometer with Cuα radiation, λ= 0.15417 nm), scanning electron microscopy (SEM, EDAX-4800), transmission electron microscopy (TEM, Japan JEOL JEM-200CX) with energy dispersive X-ray spectroscopic (EDS, EDAX-4800) results and X-ray photoelectron spectroscopy (XPS, microscopy). The BET surface areas were derived from N_2 adsorption-desorption isotherms measured using ASAP2020 (Micromeritics, United States). Thermogravimetric analysis was carried on LABSYS from SETERAM. During photocatalysis, the UV-vis absorption spectra of RhB solution at different catalytic stages were measured on a LAMBDA-35 (US)

spectrometer.

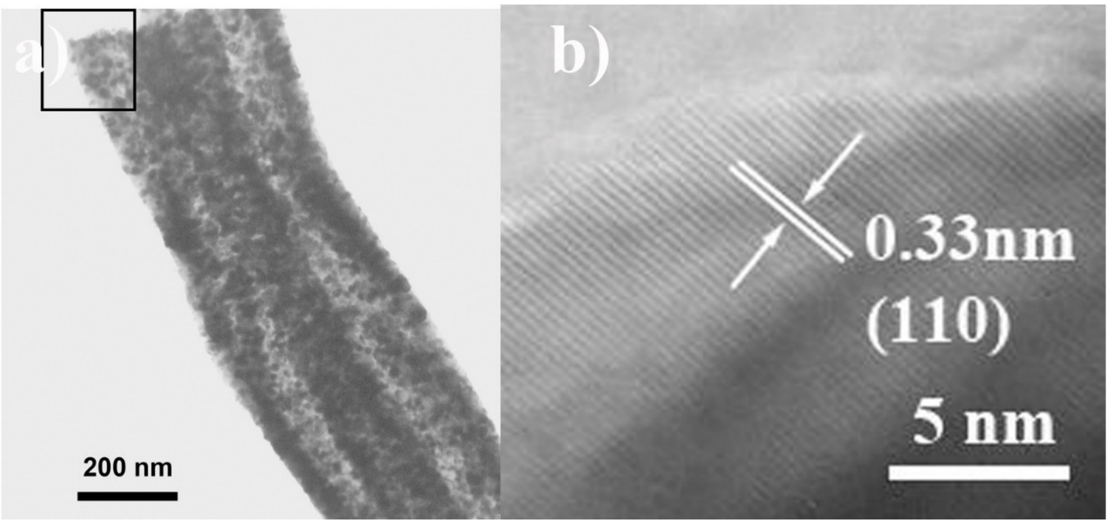

Fig. S1 (a) HRTEM images of the ATTFs-5%, (b) lattice plane of the rutile TiO_2.

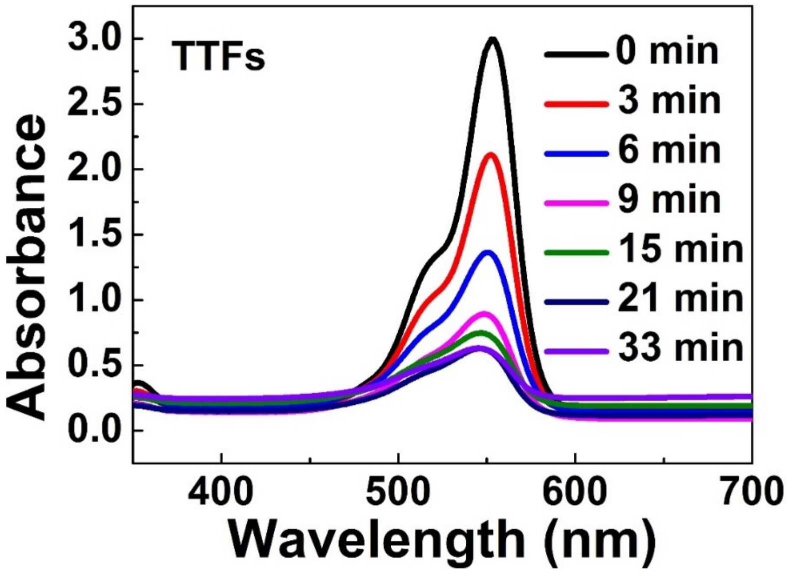

Fig. S2 RhB absorption curves of the degradation systems catalyzed by pristine TiO_2 at given reaction times.

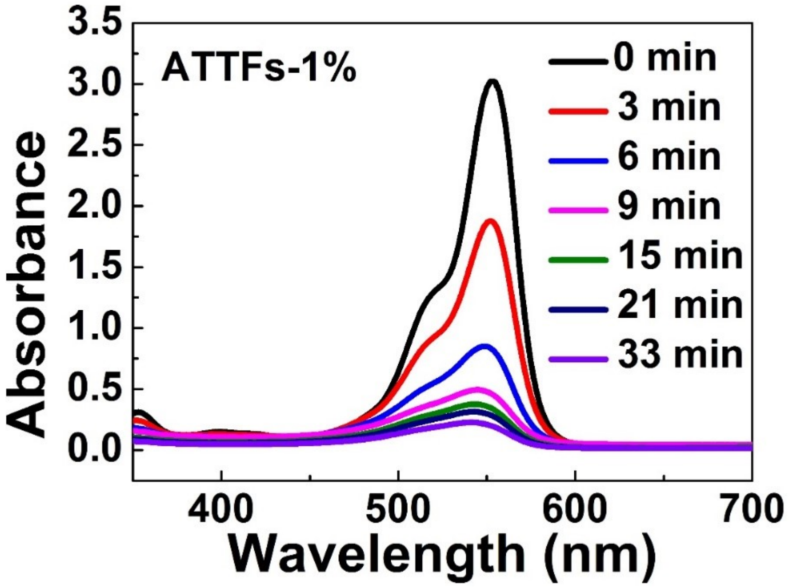

Fig. S3 RhB absorption curves of the degradation systems catalyzed by 1% Ag/TiO_2 at given reaction times.

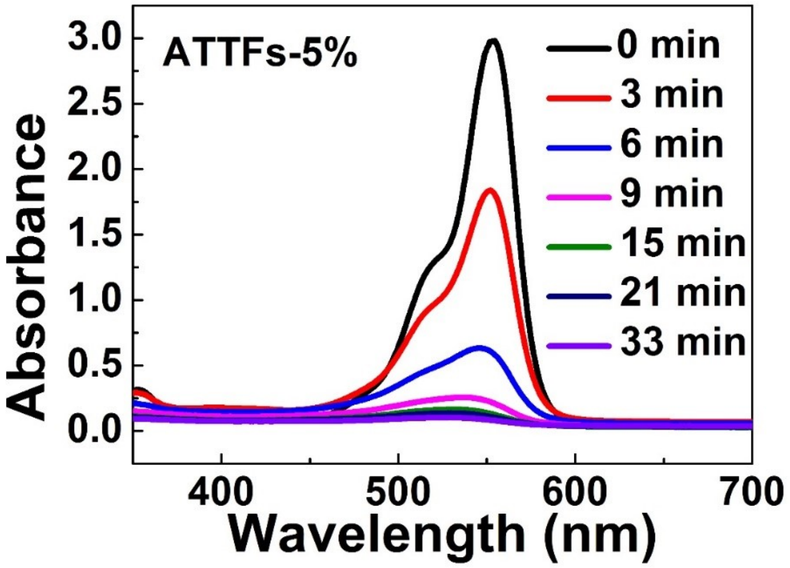

Fig. S4 RhB absorption curves of the degradation systems catalyzed by 5% Ag/TiO_2 at given reaction times.

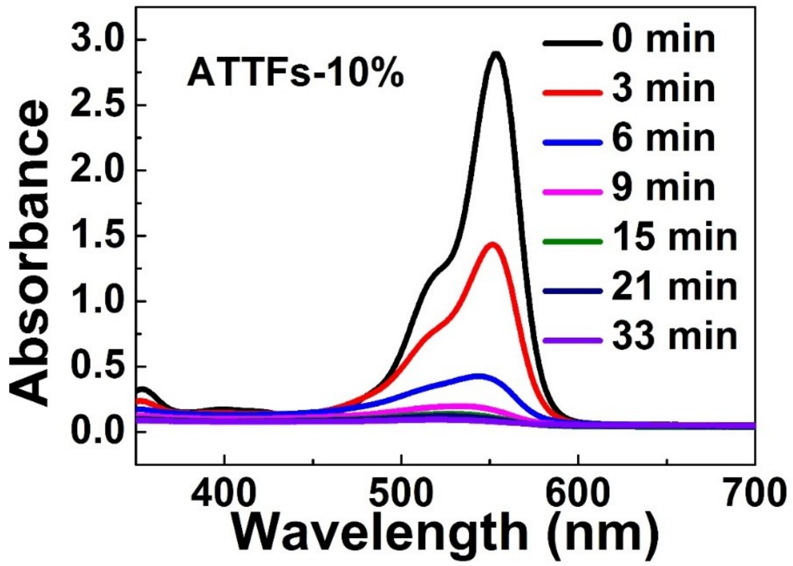

Fig. S5 RhB absorption curves of the degradation systems catalyzed by 10% Ag/TiO_2 at given reaction times.

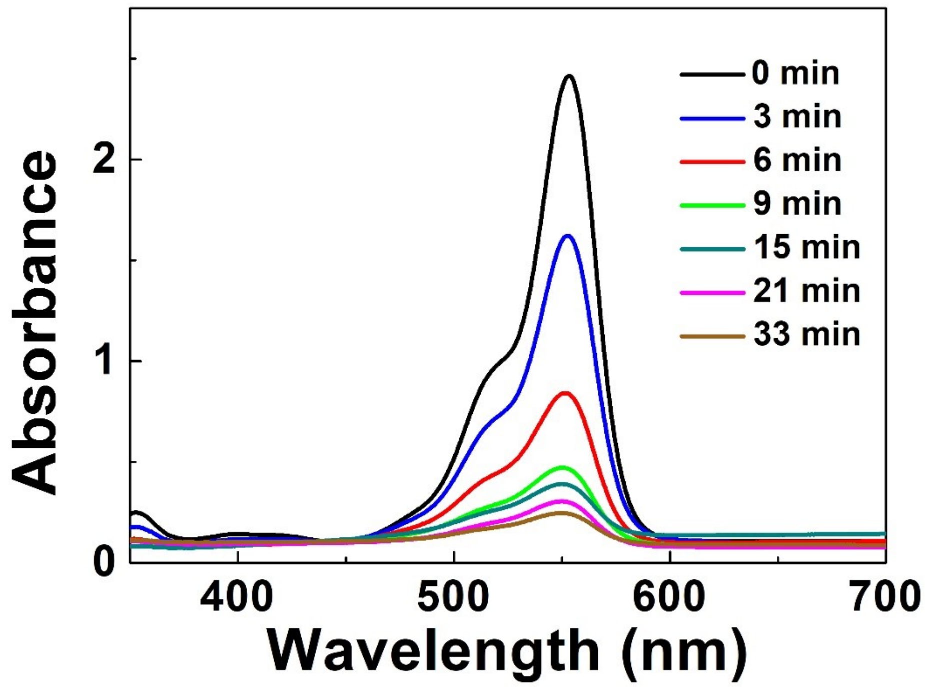

Fig. S6 RhB absorption curves of the degradation systems catalyzed by 5% Ag/commercial TiO_2 powder at given reaction times.

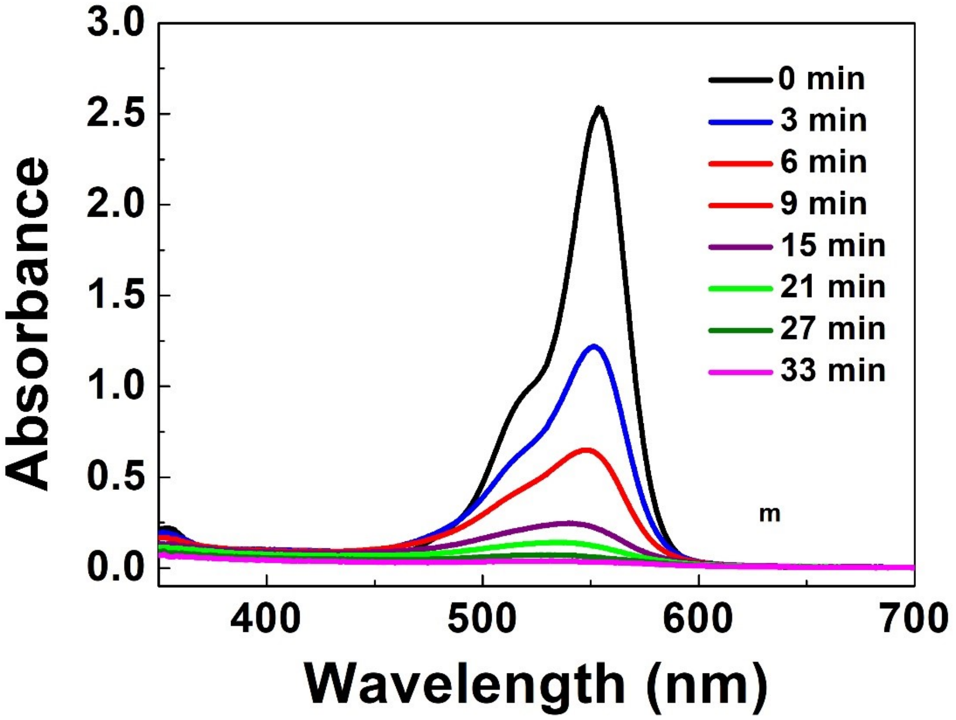

Fig. S7 RhB absorption curves of the degradation systems catalyzed by 5% Ag/TiO_2 at given reaction times under visible light irradiation by using UV filter.
